# HIV testing history and positivity among repeat testers and first-time testers in older adults: a descriptive study from Hejiang county, Southwest China, 2018–2025

**DOI:** 10.3389/fpubh.2026.1836119

**Published:** 2026-06-19

**Authors:** Yuqing Zhang, Li Zhao, Yao Luo, Chen Chen, Hong Liu, Dinglun Zhou

**Affiliations:** 1 West China School of Public Health and West China Fourth Hospital, Sichuan University, Chengdu, China; 2 Luzhou Center for Disease Control and Prevention, Luzhou, Sichuan, China; 3 Hejiang County Center for Disease Control and Prevention, Luzhou, Sichuan, China

**Keywords:** aging, HIV/aids, human immunodeficiency virus screening, positivity rate, testing history

## Abstract

**Background:**

Understanding HIV testing patterns among older adults is critical for optimizing screening strategies, yet detailed descriptions of testing history remain scarce. We leveraged data from a county-wide screening program to characterize testing patterns and HIV positivity among adults aged ≥50 years in southwestern China.

**Methods:**

We used data from a 2025 county-wide HIV screening program in Hejiang County. Records were linked to a provincial historical database (2018–2024) to classify participants as first-time or repeat testers. We compared demographics and HIV positivity rates between the two groups, and estimated the annual HIV detection rate (per 1,000 person-years) among repeat testers from 2018 to 2025. Multivariable logistic regression assessed the association between testing history and HIV positivity, adjusting for age, gender, education, and marital status. Multivariable Cox regression examined factors associated with HIV detection, with a counting process model as main analysis and a midpoint-based model as sensitivity analysis.

**Results:**

Among 235,771 tests, overall HIV positivity was 0.50% (*n* = 1,176). First-time testers (*n* = 20,438) were younger (60.2% aged 50–59 years) and more often male (57.8%), whereas repeat testers (*n* = 215,333) were older (41.5% aged ≥70 years). Positivity rates were higher among repeat testers than first-time testers (0.51 vs. 0.38%), especially in males. In multivariable logistic regression, repeat testers had higher odds of HIV positivity (aOR = 1.53, 95% CI: 1.39–1.64, *P* < 0.001). Among repeat testers, the annual detection rate rose from 0.19 (2018) to a peak of 1.32 (2021), then fell to 0.55 (2025). In multivariable Cox regression, male (HR = 2.61), age 60–69 years (HR = 1.54), unmarried (HR = 2.39), divorced (HR = 2.06), and widowed (HR = 5.65) were associated with higher hazard, while high school education or above was protective (HR = 0.36). Sensitivity analysis using a midpoint-based Cox model yielded consistent estimates (e.g., HR for male: 2.61 in both models), with all *P* < 0.05 except age ≥80 and unknown marital status.

**Conclusion:**

This descriptive study reveals marked demographic differences and HIV positivity between first-time and repeat testers aged ≥50 years, and identifies demographic factors associated with HIV detection. Linking screening data to historical records offers a practical method for characterizing testing histories and may serve as a reference for similar settings.

## 1 Introduction

HIV remains a persistent global health threat, with 40.8 million people living with HIV and 630,000 AIDS-related deaths reported in 2024 (1). A critical but often overlooked dimension of this challenge is the aging of the HIV epidemic: by 2030, more than half of people living with HIV are projected to be aged 50 years or older (2–4). A recent global burden of disease study specifically focusing on older adults further confirmed that the number of older adults living with HIV has more than doubled over the past three decades, with the fastest growth observed in East Asia (5). Yet testing strategies and surveillance systems have historically focused on younger populations, leaving older adults less well characterized (6, 7).

Challenges in understanding testing patterns among older populations are reflected in both global and local settings. Globally, UNAIDS has estimated that the total number of people older than 50 years with HIV infection increased from 5.4 million to 8.1 million between 2015 and 2020 (8). In countries with a high HIV burden, such as Kenya, 30% of people living with HIV had never been tested previously, a pattern especially common among men aged ≥40 years (9, 10). In the high-burden southwestern region of China, testing uptake is particularly low among subgroups such as rural men aged ≥50 years, with surveillance data suggesting rates below 40% (11). Despite the high and growing burden in older populations, detailed descriptions of testing history—such as who tests repeatedly vs. who remains untested—are scarce globally, and even more so at the local level (12).

Understanding the characteristics of individuals with different testing histories is a necessary first step for informing age-appropriate HIV screening strategies. Studies from diverse contexts have shown that individuals who are less engaged with testing services often differ systematically from those who test frequently, not only in actual infection risk but also in perceived risk, testing willingness, and barriers such as stigma or limited access (13). However, most existing studies focus on key populations (e.g., men who have sex with men, sex workers) or on general populations without linking individual testing histories. As a result, there is limited empirical evidence describing how testing patterns vary by age, gender, and socioeconomic factors in older adults (14, 15)—a gap that constrains the development of evidence-based, age-appropriate screening strategies.

To address this gap, we conducted a descriptive study using data from a 2025 county-wide HIV screening program among adults aged ≥50 years in Hejiang County, southwestern China – an area where HIV cases among older adults increased by more than 50% annually during 2015–2019 (16). We classified participants as first-time or repeat testers by linking records to a historical provincial database (2018–2024). This design enabled a dual approach: a cross-sectional comparison of demographic and positivity rate profiles between these groups, and a retrospective cohort analysis of repeat testers to examine longitudinal trends in detection rate over an 8-year period using person-years. By focusing on an older population and systematically characterizing testing history, this study provides a descriptive baseline that may serve as a reference for similar settings seeking to understand testing patterns in aging populations.

## 2 Methods

### 2.1 Implementation of the HIV expanded screening program (2025)

A population-wide HIV screening initiative was implemented for all adults aged 50 years and older in Hejiang County, Sichuan Province, from January 1 to September 1, 2025. The program was coordinated by the county-level public health authority and executed through the existing multi-tiered healthcare service network.

To facilitate systematic implementation, a targeted sampling frame was established prior to the campaign. This registry was developed by integrating administrative health records with verified community-based household lists to enable structured mobilization and coverage tracking.

HIV testing was delivered through a multi-channel, client-centered strategy to maximize accessibility and uptake. The core components included:

(1) Facility-based testing: Standard provider-initiated testing and counseling was available at designated public health clinics;

(2) Routine service integration: An opt-out HIV screening approach was incorporated into general health services (e.g., chronic disease management, routine check-ups) for all eligible individuals;

(3) Community-based outreach: Mobile testing stations were set up at organized community events to provide confidential, opportunistic screening;

(4) Home-based testing: upon request, confidential testing was offered at home for individuals reporting mobility constraints or strong privacy preferences.

The study protocol was approved by the Ethics Committee of West China Fourth Hospital and West China School of Public Health, Sichuan University (Approval No. Gwll2022063), which waived the requirement for informed consent because the study involved only retrospective analysis of de-identified routine surveillance data, posing minimal risk to participants.

### 2.2 Laboratory procedures: sample collection, testing, and case confirmation

All testing procedures strictly adhered to the National Diagnostic Criteria for HIV/AIDS (WS 293-2019) and followed the recommended algorithm outlined in the National Guideline for Detection of HIV/AIDS (2025) (17).

Venous or finger-prick whole blood samples were collected by trained personnel using aseptic technique. HIV testing followed a standardized two-step algorithm: an initial screening phase followed by a confirmatory phase for reactive specimens.

#### 2.2.1 Initial screening

All specimens were initially tested using a high-sensitivity fourth-generation HIV-1/2 antigen/antibody combined enzyme immunoassay (Wantai HIV Ag/Ab ELISA Kit; Beijing Wantai Biological Pharmacy Enterprise Co., Ltd., China). All reactive samples were immediately retested in duplicate using the same assay. Specimens that were repeatedly reactive in the screening assay were classified as preliminarily positive and referred for confirmatory testing.

#### 2.2.2 Confirmatory testing

All preliminarily positive specimens were transferred to the central reference laboratory at the Luzhou Center for Disease Control and Prevention for definitive confirmation. Confirmation was performed using a licensed Western blot assay (HIV Blot 2.2; MP Biomedicals) according to the manufacturer's protocol. In cases where Western blot results were indeterminate or required further validation, supplementary nucleic acid testing (NAT) was performed using either the COBAS^®^ TaqMan HIV-1 Test v2.0 (Roche molecular systems) or the m2000 RealTime HIV-1 Assay (Abbott Laboratories).

A confirmed HIV-positive case was strictly defined by a positive result from both the initial screening assay and the supplemental confirmatory assay(s). All testing was conducted under established internal and external quality control procedures.

### 2.3 Data processing and study population

Individual-level data were obtained from the 2025 county-wide HIV screening program targeting adults aged ≥50 years in Hejiang County. Records were excluded if individuals lacked local household registration (because the historical testing database 2018–2024 only covered registered residents, making linkage otherwise impossible), had missing core demographic information (age or gender), or had an invalid unique personal identifier.

The cleaned 2025 records were then deterministically linked to the county's historical HIV testing database (January 1, 2018–December 31, 2024) using the unique personal identifier. Based on this linkage, participants were classified into two mutually exclusive groups for cross-sectional analysis:

- Repeat testers: individuals with at least one recorded HIV test in the historical database prior to 2025;
- First-time testers: individuals with no matching record in the historical database, indicating no documented HIV test in the county within the available historical window (since January 1, 2018).

**Figure 1:**
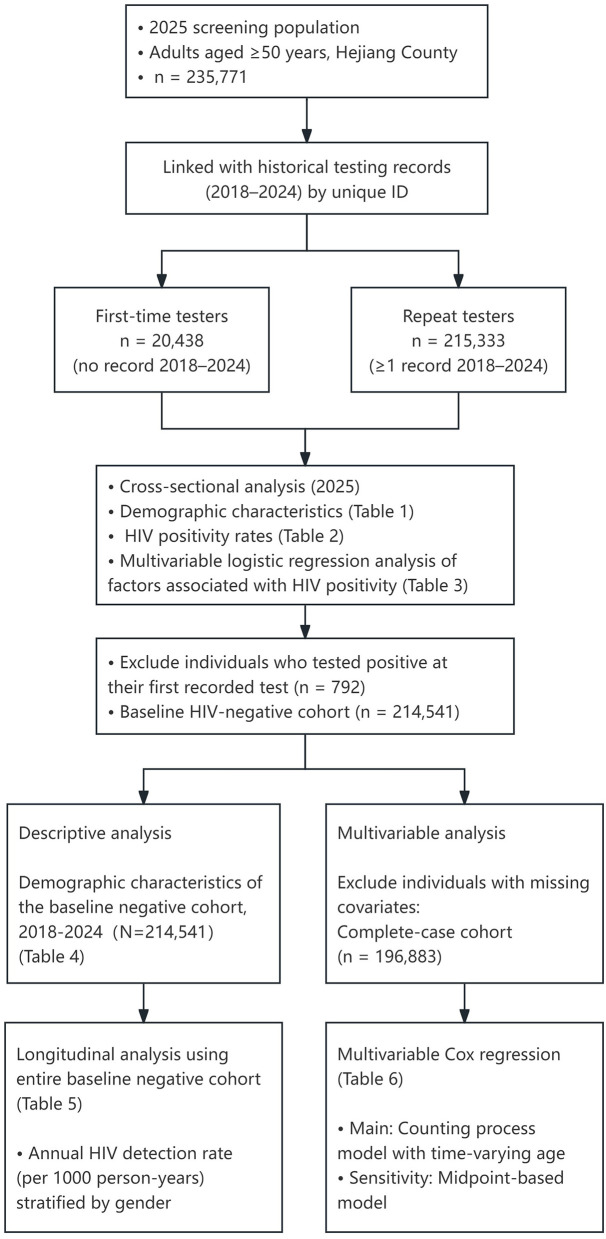
Study population selection and analysis flow.

For longitudinal analysis, a retrospective cohort was constructed from all repeat testers. Their complete historical testing records (2018–2024) were retrieved to establish individual testing trajectories. Individuals whose earliest recorded test indicated a pre-existing HIV-positive diagnosis were excluded, as they were not at risk for a new diagnosis. The final analytic cohort for longitudinal assessment thus consisted of repeat testers who were HIV-negative at baseline (earliest recorded test between 2018 and 2024). Figure 1 summarizes the study population selection process. All data were anonymized, and test results were managed with strict confidentiality in accordance with ethical guidelines.

### 2.4 Definition and calculation of indicators

#### 2.4.1 Cross-sectional analysis (2025 screening)

For cross-sectional analyses, each individual's most recent test record from the 2025 program was used. Age was calculated as a continuous variable from the date of birth to the testing date. The positivity rate for any subgroup was defined as the proportion of unique individuals with a confirmed positive result among all unique individuals tested in that subgroup, expressed as a percentage.

#### 2.4.2 Longitudinal analysis (repeat testers cohort, 2018–2025)

For the retrospective cohort of repeat testers, the primary outcome was the first confirmed HIV-positive test recorded between January 1, 2018, and September 1, 2025.

The annual HIV detection rate was calculated for each calendar year from 2018 to 2025. The numerator was the number of individuals whose first positive test occurred in year Y. The denominator was the person-years contributed by individuals at risk in year Y, defined as those who had at least one HIV test recorded in year Y and were HIV-negative prior to year Y (i.e., all available historical test results before Y were negative). Person-years were calculated as the sum of observation time from the first negative test of the year until the earliest of: HIV diagnosis, last negative test of the year, or end of the study (September 1, 2025). The detection rate was expressed per 1,000 person-years with 95% confidence intervals estimated using the exact Poisson method.

### 2.5 Statistical analysis

All statistical analyses were conducted using R software (version 4.3.2). Categorical variables were summarized as frequencies and percentages. Differences in demographic and clinical characteristics between first-time and repeat testers were assessed using the chi-square test or Fisher's exact test, as appropriate. Given the exploratory nature of multiple group comparisons, unadjusted P-values are reported in the primary analysis; the implications of multiple testing are further considered in the discussion.

For the cross-sectional analysis of the 2025 screening data, multivariable logistic regression was used to evaluate the independent association between testing history (first-time vs. repeat) and HIV positivity, adjusting for age group, gender, education, and marital status. Adjusted odds ratios (aOR) with 95% confidence intervals were calculated. Complete-case analysis was applied, excluding individuals with missing data on any covariate.

Multivariable survival analysis was performed to identify factors associated with HIV detection. Two complementary Cox regression models were used:

- Main model: A counting process Cox model (Andersen-Gill) with time-varying age and robust standard errors clustered by individual. This model uses all available test intervals and allows age to change over time.
- Sensitivity analysis: A midpoint-based Cox model using baseline age, where the event time was imputed as the midpoint between the last negative and first positive test (or the last negative date for censored individuals).

The proportional hazards assumption was assessed using Schoenfeld residuals for the midpoint Cox model; the global test was not significant (*P* = 0.248), indicating that the assumption was not violated. Model discrimination for the counting process model was evaluated using the concordance index (C-index). All tests were two-sided, and a *P* value < 0.05 was considered statistically significant.

### 2.6 Data completeness assessment

We examined missing data patterns for key covariates (gender, age, education, and marital status). Individuals with missing data on any of these covariates were excluded from multivariable analyses (complete-case analysis). Missingness was most notable for education (6.51%) and marital status (3.94%). A comparison of complete and incomplete cases is provided in Supplementary Table S1, and the distribution of missing education and marital status by age group is shown in Supplementary Table S2.

## 3 Results

### 3.1 Cross-sectional findings from the 2025 screening program

#### 3.1.1 Basic characteristics of the screened population

In 2025, a total of 235,771 HIV tests were conducted among individuals aged 50 years and older in Hejiang County. Among those tested, 215,333 were repeat testers, and 20,438 were first-time testers. A total of 1,176 individuals tested positive, with an overall positivity rate of 0.50%.

#### 3.1.2 Comparison of demographic characteristics between repeat and first-time testers

Significant demographic differences were observed between first-time and repeat testers (all *P* < 0.001; Table 1). First-time testers were more likely to be male (57.81 vs. 46.27%) and were predominantly aged 50–59 years (60.16%). In contrast, the repeat-testing group was older, with a notably higher proportion aged ≥70 years (41.46%). Marital status also differed: a slightly higher percentage of first-time testers were married, whereas repeat testers had significantly higher proportions of divorced and widowed individuals. Educational attainment was relatively higher among first-time testers, with 32.87% having completed junior high school or above, compared to 23.85% of repeat testers. Correspondingly, the proportion classified as “illiterate or semi-illiterate” was lower among first-time testers (32.33 vs. 39.54%).

**Table 1:** Demographic characteristics of first-time and repeat testers, Hejiang County, 2025.

Characteristic	Repeat testers (*n* = 215,333)	First-time testers (*n* = 20,438)	χ^2^	*P*-value
Gender				
Male	99,634 (46.27)	11,815 (57.81)	997.17	< 0.001
Female	115,699 (53.73)	8,623 (42.19)		
Age group				
50–59	65,774 (30.55)	12,296 (60.16)	8,463.65	< 0.001
60–69	60,270 (27.99)	5,174 (25.32)		
70–79	65,127 (30.24)	2,056 (10.06)		
≥80	24,162 (11.22)	912 (4.46)		
Marital status				
Unmarried	6,679 (3.10)	384 (1.88)	2,403.34	< 0.001
Married	172,737 (80.22)	17,455 (85.40)		
Widowed	30,085 (13.97)	575 (2.81)		
Divorced	3,214 (1.49)	250 (1.22)		
Unknown^*^	1,013 (0.47)	321 (1.57)		
Missing^*^	1,605 (0.75)	1,453 (7.11)		
Education level				
Illiterate or semi-illiterate	85,135 (39.54)	6,607 (32.33)	1,468.45	< 0.001
Primary school	67,954 (31.56)	6,376 (31.20)		
Junior high school	43,320 (20.12)	6,341 (31.03)		
Senior high school or above	8,025 (3.73)	376 (1.84)		
Missing^*^	10,899 (5.06)	738 (3.61)		
**Total**	215,333 (100.00)	20,438 (100.00)		

^*^“Unknown” indicates a recorded marital status of “Unknown” (code 90); “Missing” indicates no recorded marital status. Education level was missing for a small proportion of individuals, as shown in the table. For each χ^2^ test, missing data were excluded; for gender, the uncorrected Pearson χ^2^ is reported.

#### 3.1.3 Screening positivity rates by age group and gender

The overall screening positivity rate in 2025 was significantly higher among repeat testers than among first-time testers (0.51 vs. 0.38%; RR = 1.34, 95% CI: 1.06–1.70; χ^2^ = 5.932, *P* = 0.015). This pattern varied by gender and age (Table 2).

**Table 2:** HIV positivity rates by age group and gender among first-time and repeat testers, 2025.

Characteristic	Repeat testers			First-time testers			χ^2^	*P*-value
	Total	Number positive	Positivity rate (%)	Total	Number positive	Positivity rate (%)		
Male								
50–59	28,395	154	0.54	7,179	24	0.33	16.218	< 0.001
60–69	28,939	227	0.78	3,114	20	0.64		
70–79	31,279	316	1.01	1,162	8	0.69		
≥80	11,021	87	0.79	360	1	0.28		
Total	99,634	784	0.79	11,815	53	0.45		
Female								
50–59	37,379	75	0.20	5,117	14	0.27	0.1013	0.750
60–69	31,331	109	0.35	2,060	6	0.29		
70–79	33,848	111	0.33	894	4	0.45		
≥80	13,141	19	0.14	552	1	0.18		
Total	115,699	314	0.27	8,623	25	0.29		
**Total**	215,333	1,098	0.51	20,438	78	0.38		

^*^χ^2^ test comparing positivity rates between repeat testers and first-time testers within each gender.

A marked disparity was observed among males. The positivity rate was 1.75 times higher in repeat testers (0.79%) than in first-time testers (0.45%) (RR = 1.75, 95% CI: 1.33–2.32). This elevated rate among repeat-tested males was particularly pronounced in the 70–79 years age group (1.01%). In contrast, positivity among first-time testers was consistently lower across all age groups and showed no clear age-dependent trend.

Among females, overall positivity rates were comparable between the two groups. However, in the 50–59 and 70–79 years age groups, positivity was marginally higher among first-time testers than among repeat testers.

#### 3.1.4 Multivariable logistic regression analysis of factors associated with HIV positivity

To assess the independent association between testing history and HIV positivity while adjusting for potential confounders, we performed multivariable logistic regression including testing history, age group, gender, education, and marital status (Table 3). After excluding individuals with missing covariates (mainly education or marital status), 224,413 participants (95.2% of the 2025 screened population) were included in the complete-case analysis.

**Table 3:** Multivariable logistic regression analysis of factors associated with HIV positivity among adults aged ≥50 years, Hejiang County, 2025 (*n* = 224,413^*^).

Variable	Multivariable logistic regression analysis	
	aOR (95% CI)	*P*
Testing history		
First-time	1.00 (ref)	
Repeat	1.53 (1.39–1.64)	< 0.001
Gender		
Female	1.00 (ref)	–
Male	2.40 (2.09–2.77)	< 0.001
Age group		
50–59	1.00 (ref)	–
60–69	2.33 (1.98–2.75)	< 0.001
70–79	3.16 (2.65–3.78)	< 0.001
≥80	1.73 (1.33–2.23)	< 0.001
Education		
Illiterate or semi-illiterate	1.00 (ref)	–
Primary school	0.69 (0.60–0.79)	< 0.001
Junior high school	0.42 (0.35–0.51)	< 0.001
Senior high school or above	0.59 (0.37–0.87)	0.0128
Marital status		
Married	1.00 (ref)	–
Unmarried	4.25 (3.45–5.21)	< 0.001
Divorced	8.09 (6.25–10.34)	< 0.001
Widowed	3.70 (3.12–4.38)	< 0.001
Unknown^*^	0.44 (0.13–1.04)	0.1055

^*^Use complete cases. Adjusted for all variables listed in the table. Unknown marital status accounted for < 0.5% of the sample; the wide confidence interval reflects low precision.

Repeat testers had significantly higher odds of HIV positivity than first-time testers (adjusted odds ratio [aOR] = 1.53, 95% CI: 1.39–1.64, *P* < 0.001). Male gender, older age (especially 60–79 years), unmarried, divorced, or widowed status, and lower educational attainment were also independently associated with higher odds (all *P* < 0.05; Table 3). Unknown marital status was not significantly associated (aOR = 0.44, 95% CI: 0.13–1.04, *P* = 0.106).

### 3.2 Longitudinal findings from the repeat-tester cohort (2018–2025)

#### 3.2.1 Baseline characteristics of the HIV-negative repeat tester cohort

The demographic characteristics of the baseline HIV-negative repeat tester cohort (*n* = 214,541) are presented in Supplementary Table S3. The cohort was predominantly female (53.78%) and older, with 60.27% aged ≥60 years. A substantial proportion had low educational attainment (30.14% illiterate or semi-illiterate) and were married (78.68%). Missing data accounted for 6.51% for education and 3.94% for marital status.

#### 3.2.2 Trends in detection rate among the repeat-tested population

In this cohort, the annual HIV detection rate (per 1,000 person-years) increased from 0.19 (95% CI: 0.07–0.40) in 2018 to a peak of 1.32 (95% CI: 1.12–1.56) in 2021, before gradually declining to 0.55 (95% CI: 0.45–0.66) in 2025 (Table 4). Detection rates were consistently higher among males than females throughout the observation period (Table 4).

**Table 4:** Annual HIV detection rate (per 1,000 person-years) among repeat testers who were HIV-negative at baseline, 2018–2025.

	Male			Female			Total		
Variable	Newly identified positive (*n*)	Person-years	Detection rate per 1,000 person-years (95% CI)	Newly identified positive (*n*)	Person-years	Detection rate per 1,000 person-years (95% CI)	Newly identified Positive (*n*)	Person-years	Detection rate per 1,000 person-years (95% CI)
**2018**	5	15,494.44	0.32 (0.10,0.75)	1	16,827.85	0.06 (0.00,0.33)	6	32,322.29	0.19 (0.07,0.40)
**2019**	33	38,603.7	0.85 (0.59,1.20)	25	53,291.31	0.47 (0.30,0.69)	58	91,895.01	0.63 (0.48,0.82)
**2020**	60	43,651.94	1.37 (1.05,1.77)	30	54,495.89	0.55 (0.37,0.79)	90	98,147.83	0.92 (0.74,1.13)
**2021**	81	46,926.13	1.73 (1.37,2.15)	66	64,115.36	1.03 (0.80,1.31)	147	111,041.49	1.32 (1.12,1.56)
**2022**	66	49,701.14	1.33 (1.03,1.69)	36	64,839.66	0.56 (0.39,0.77)	102	114,540.8	0.89 (0.73,1.08)
**2023**	75	57,862.04	1.30 (1.02,1.62)	46	73,552.31	0.63 (0.46,0.83)	121	131,414.35	0.92 (0.76,1.10)
**2024**	73	79,331.1	0.92 (0.72,1.16)	36	95,326.55	0.38 (0.26,0.52)	109	174,657.65	0.62 (0.51,0.75)
**2025**	86	98,646.73	0.87 (0.70,1.08)	31	115,185.73	0.27 (0.18,0.38)	117	213,832.46	0.55 (0.45,0.66)

^*^Person-years represent the total observation time contributed by individuals in that year. Due to right censoring and event occurrence, person-years may be less than the number of individuals tested in that year.

#### 3.2.3 Multivariable analysis of factors associated with HIV detection

For multivariable analysis, we further excluded 17,658 individuals (8.2% of the baseline cohort) with missing covariates or abnormal gender code (gender = 9), yielding a final analytic sample of 196,883 individuals (Supplementary Tables S1, S2; S1 compares complete vs. incomplete cases, S2 shows missingness by age group). Multivariable Cox regression models were used to assess factors associated with HIV diagnosis (first positive test). The main analysis employed a counting process Cox model with time-varying age and robust standard errors clustered by individual, while a midpoint-based Cox model using baseline age was performed as a sensitivity analysis. Results from both models were highly consistent (Table 5).

**Table 5:** Multivariable associations between selected characteristics and HIV detection among adults aged ≥50 years, Hejiang County, 2018–2025.

Variable	Main analysis (counting process Cox)		Sensitivity analysis (midpoint Cox)	
	HR (95% CI)	*P*	HR (95% CI)	*P*
Gender				
Female	1.00 (ref)	< 0.001	1.00 (ref)	< 0.001
Male	2.61 (2.06–3.30)		2.61 (2.07–3.29)	
Age group				
50–59	1.00 (ref)	–	1.00 (ref)	–
60–69	1.54 (1.19–1.99)	< 0.001	1.37 (1.07–1.75)	0.012
70–79	1.31 (0.94–1.82)	0.108	1.08 (0.79–1.47)	0.638
≥80	1.16 (0.64–2.10)	0.635	0.89 (0.50–1.56)	0.680
Education				
Illiterate or semi-illiterate	1.00 (ref)	–	1.00 (ref)	–
Primary school	1.06 (0.84–1.34)	0.639	1.10 (0.87–1.38)	0.430
Junior high school	0.84 (0.60–1.17)	0.298	0.81 (0.59–1.11)	0.186
Senior high school or above	0.36 (0.13–0.98)	0.046	0.36 (0.13–0.97)	0.044
Marital status				
Married	1.00 (ref)	–	1.00 (ref)	–
Unmarried	2.39 (1.66–3.44)	< 0.001	2.38 (1.66–3.42)	< 0.001
Divorced	2.06 (1.51–2.81)	< 0.001	2.24 (1.68–3.00)	< 0.001
Widowed	5.65 (3.72–8.59)	< 0.001	5.85 (3.83–8.92)	< 0.001
Unknown	1.61 (0.66–3.93)	0.298	1.13 (0.46–2.73)	0.793

In the main model, male (HR = 2.61, 95% CI: 2.06–3.30, *P* < 0.001) and age 60–69 years (HR = 1.54, 95% CI: 1.19–1.99, *P* < 0.001) were associated with increased hazard of HIV diagnosis, while age 70–79 and ≥80 years were not statistically significant in this model. Higher education (senior high school or above) was protective (HR = 0.36, 95% CI: 0.13–0.98, *P* = 0.046). Compared with married individuals, unmarried (HR = 2.39), divorced (HR = 2.06), and widowed (HR = 5.65) statuses were associated with significantly higher hazard (all *P* < 0.001). The sensitivity analysis yielded nearly identical estimates (e.g., for male: HR = 2.61, 95% CI: 2.07–3.29; for senior high school: HR = 0.36, 95% CI: 0.13–0.97). The proportional hazards assumption was met for the midpoint model (global test *P* = 0.248), although age group showed a marginal deviation (*P* = 0.046); this was addressed by using a time-varying age model as the main analysis, which does not require the assumption.

## 4 Discussion

This study integrated cross-sectional data from a 2025 county-wide HIV screening program with longitudinal follow-up of a repeat-testing cohort to describe testing patterns and HIV positivity among adults aged ≥50 years in an area with a high HIV burden in southwestern China.

We observed a significantly higher HIV positivity rate among repeat testers (0.51%) than among first-time testers (0.38%), a difference that was particularly pronounced in males. Among repeat testers, the annual HIV detection rate declined from a peak of 1.32 per 1,000 person-years in 2021 to 0.55 per 1,000 person-years in 2025. These descriptive findings highlight distinct testing histories and positivity profiles that may inform the interpretation of routine screening data.

Marked demographic differences between first-time and repeat testers were evident. First-time testers were younger (60.2% aged 50–59 years) and had higher educational attainment, while repeat testers were older (41.5% aged ≥70 years). These patterns are consistent with studies suggesting that individuals who are less engaged with testing services may have lower perceived risk or face structural barriers (13, 18, 19). In the Chinese context, fear of stigma and discrimination may also influence testing initiation among those who do not perceive themselves as high-risk (20, 21). Women were more likely to be repeat testers, possibly reflecting greater health-seeking behavior or more frequent engagement with health services (22, 23). Studies confirm that women, particularly in older age groups or with higher education, exhibit greater acceptance of voluntary testing (24, 25). Notably, HIV positivity rates among women did not differ significantly between first-time and repeat testers, suggesting that HIV positivity was similarly distributed across testing history groups among women, which may indicate more balanced detection across risk strata.

The higher positivity rate observed among repeat testers, especially older males, is consistent with the possibility that this group has had a longer cumulative observation period in the testing system (26, 27). The concentration of detections in the 70–79 year age group across both cohorts aligns with this pattern. Among repeat testers, the annual detection rate increased from 2018 to a peak in 2021 and then declined steadily to 0.55 per 1,000 person-years by 2025. This decline coincided with a period when screening efforts were scaled up, including the intensive 2025 program. While the decline could reflect a reduction in new detections, it may also be influenced by screening saturation (most individuals in this cohort had been tested repeatedly) or by changes in testing frequency over time.

The contrasting characteristics of first-time and repeat testers illustrate that the population reached by routine screening is not homogeneous. Relying solely on data from repeat testers may overrepresent individuals who have been tested frequently and have higher positivity rates, whereas first-time testers represent a different segment of the population. Similar patterns have been observed elsewhere: in Kenya, expanded testing eligibility increased HIV diagnoses, yet the proportion of first-time testers remained substantial, especially among older men (9). These observations underscore the importance of distinguishing testing history in routine surveillance. In our study, subgroups such as older males, widowed/divorced individuals, and those with lower education had higher positivity among repeat testers—findings that may inform targeted screening efforts. Acknowledging this heterogeneity is important when interpreting cross-sectional screening data and when designing strategies to reach underserved groups. The approach of linking screening records to historical testing data to classify individuals by testing history could be implemented in other settings to monitor the representativeness of their own screening programs.

Several limitations should be considered. First, the classification of first-time testers relied on historical records available from 2018 onward; individuals tested before 2018 may have been misclassified. However, the younger age profile of first-time testers suggests that such misclassification is unlikely to substantially affect the main descriptive patterns. Second, we could not distinguish between new infections and long-standing infections diagnosed for the first time; the detection rate therefore reflects diagnoses, not incidence. Third, the study was conducted in a single county in a high-prevalence region, which may limit generalizability to other populations. Nevertheless, the detailed characterization of testing patterns provides a useful reference for similar settings. Fourth, we lacked data on behavioral risk factors (e.g., sexual behavior, drug use), which could offer further insight into the observed patterns. Fifth, excluding individuals without local household registration may have introduced selection bias, as this migrant population might have different HIV risk profiles and testing behaviors; thus, our findings are most generalizable to the registered resident population. Despite these limitations, the large sample size, linkage with historical records, and rigorous analytical approach support the robustness of our conclusions.

In this descriptive study of a county-wide HIV screening program among adults aged ≥50 years, first-time and repeat testers differed markedly in demographic characteristics and HIV positivity. Among repeat testers, the annual detection rate declined after 2021. These findings illustrate the importance of considering testing history when interpreting screening data and may serve as a reference for similar settings seeking to characterize testing patterns in aging populations.

## Data Availability

The data analyzed in this study is subject to the following licenses/restrictions: Data are not publicly available due to privacy and ethical restrictions. Access may be granted upon reasonable request to the corresponding author, subject to IRB approval. Requests to access these datasets should be directed to Dinglun Zhou, zhoudinglun@scu.edu.cn.

## References

[1] UNAIDS. FACT SHEET 2025: Global HIV statistics. Geneva: Joint United Nations Programme on HIV/AIDS (2025). Available online at: https://www.unaids.org/sites/default/files/media_asset/UNAIDS_FactSheet_en.pdf

[2] Carter A Walters MK Jahagirdar D Brewer ED Novotney A Lasher D . Lancet HIV. (2024) 11:e807–22. doi: 10.1016/S2352-3018(24)00212-1 39608393 PMC11612058

[3] O'Connor SM Song R Viguerie A Buchacz K Lyles CM Farnham PG . Projected increases in older people with HIV in the United States through 2040. Open Forum Infect Dis. (2025) 12:ofaf656. doi: 10.1093/ofid/ofaf656 41262359 PMC12626220

[4] Marty L Diawara Y Rachas A Grabar S Costagliola D Supervie V. Projection of age of individuals living with HIV and time since ART initiation in 2030: estimates for France. J Int AIDS Soc. (2022) 25:e25986. doi: 10.1002/jia2.25986 36176023 PMC9523002

[5] Fu L Tian T Wang B Lu Z Bian J Zhang W . Global, regional, and national burden of HIV and other sexually transmitted infections in older adults aged 60-89 years from 1990 to 2019: results from the global burden of disease study 2019. The lancet Healthy longevity. (2024) 5:e17–30. doi: 10.1016/S2666-7568(23)00214-3 38183996

[6] Ekong N Curtis H Ong E Sabin CA Chadwick D; British HIV Association (BHIVA) Audit and Standards Sub-Committee. Monitoring of older HIV-1-positive adults by HIV clinics in the United Kingdom: a national quality improvement initiative. HIV Med. (2020) 21:409–17. doi: 10.1111/hiv.12842 32125760

[7] McCullagh C. Policies relating to prevention and detection of HIV/AIDS in older adults: a systematic review. Gerontol. (2016) 56:556. doi: 10.1093/geront/gnw162.2237

[8] Justice AC Goetz MB Stewart CN Hogan BC Humes E Luz PM . Delayed presentation of HIV among older individuals: a growing problem. The lancet HIV. (2022) 9:e269–80. doi: 10.1016/S2352-3018(22)00003-0 35218732 PMC9128643

[9] De Anda S Njoroge A Njuguna I Dunbar MD Abuna F Macharia P . Predictors of first-time and repeat HIV testing among HIV-positive individuals in Kenya. Journal of acquired immune deficiency syndromes (1999). (2020) 85:399–407. doi: 10.1097/QAI.0000000000002469 33136736

[10] Joseph RH Musingila P Miruka F Wanjohi S Dande C Musee P . Expanded eligibility for HIV testing increases HIV diagnoses-a cross-sectional study in seven health facilities in western Kenya. PLoS One. (2019) 14:e0225877. doi: 10.1371/journal.pone.0225877 31881031 PMC6934319

[11] Gao D Zou Z Dong B Zhang W Chen T Cui W . Secular trends in HIV/AIDS mortality in China from 1990 to 2016: Gender disparities. PLoS ONE. (2019) 14:e0219689. doi: 10.1371/journal.pone.0219689 31318900 PMC6638923

[12] Lin C Casavant I Jaramillo A Green T Matovu JK. Using repeated home-based HIV testing services to reach and diagnose HIV infection among persons who have never tested for HIV, Chókwè health demographic surveillance system, Chókwè district, Mozambique, 2014–2017. PLoS ONE. (2020) 15:e0242281. doi: 10.1371/journal.pone.0242281 33216773 PMC7678994

[13] Evangeli M Pady K Wroe AL. Which psychological factors are related to HIV testing? a quantitative systematic review of global studies. AIDS and behavior. (2016) 20:880–918. doi: 10.1007/s10461-015-1246-0 26566783 PMC4799267

[14] Gebremeskel AT Gunawardena N Omonaiye O Yaya S jeihooni AK. Sex differences in HIV testing among older adults in Sub-Saharan Africa: a systematic review. Biomed Res Int. (2021) 2021:5599588. doi: 10.1155/2021/5599588 34513993 PMC8427674

[15] Schatz E Houle B Mojola SA Angotti N Williams J. How to “live a good life”: aging and HIV testing in rural South Africa. J Aging Health. (2019) 31:709–32. doi: 10.1177/0898264317751945 29318924 PMC6027599

[16] Zhou M Wang L Shao M . Epidemiological characteristics of HIV/AIDS in Luzhou City, Sichuan Province from 2015 to 2019. Henan J Prev Med. (2021) 32:857–60.

[17] National Center for AIDS/STD Control and Prevention CCfDCaP. National Guideline for Detection of HIV/AIDS (2025 Revision). In: National Center for AIDS/STD Control and Prevention CCfDCaP, editor. 2025.

[18] Jha S Gee H Coomarasamy A. Women's attitudes to HIV screening in pregnancy in an area of low prevalence. BJOG Int J Obstet Gynaecol. (2003) 110:145–8. doi: 10.1046/j.1471-0528.2003.02073.x 12618158

[19] Rou K Guan J Wu Z Li L Rotheram MJ Detels R . Demographic and behavioral factors associated with HIV testing in China. J Acquir Immune Defic Syndr. (1999) 50:432–4. doi: 10.1097/QAI.0b013e3181946088 19322039 PMC2740799

[20] Pool R Nyanzi S Whitworth JA. Attitudes to voluntary counselling and testing for HIV among pregnant women in rural south-west Uganda. AIDS Care. (2001) 13:605–15. doi: 10.1080/09540120120063232 11571007

[21] Teklehaimanot HD Teklehaimanot A Yohannes M Biratu D. Factors influencing the uptake of voluntary HIV counseling and testing in rural Ethiopia: a cross sectional study. BMC Public Health. (2016) 16:239. doi: 10.1186/s12889-016-2918-z 26955869 PMC4784416

[22] Teklehaimanot HD Teklehaimanot A. Human resource development for a community-based health extension program: a case study from Ethiopia. Hum Resour Health. (2013) 11:39. doi: 10.1186/1478-4491-11-39 23961920 PMC3751859

[23] Starrs AM Ezeh AC Barker G Basu A Bertrand JT Blum R . Accelerate progress-sexual and reproductive health and rights for all: report of the Guttmacher-Lancet Commission. Lancet. (2018) 391:2642–92. doi: 10.1016/S0140-6736(18)30293-9 29753597

[24] Ho CF Loke AY. Pregnant women's decisions on antenatal HIV screening in Hong Kong. AIDS Care. (2003) 15:821–7. doi: 10.1080/09540120310001618667 14617503

[25] Mahmoud MM Nasr AM Gassmelseed DEA Abdalelhafiz MA Elsheikh MA Adam I. Knowledge and attitude toward HIV voluntary counseling and testing services among pregnant women attending an antenatal clinic in Sudan. J Med Virol. (2007) 79:469–73. doi: 10.1002/jmv.20850 17385672

[26] Jaul E Barron J. Age-related diseases and clinical and public health implications for the 85 years old and over population. Frontiers in public health. (2017) 5:335. doi: 10.3389/fpubh.2017.00335 29312916 PMC5732407

[27] Zhang Y Fuller-Thomson E Anne MC Zhang X. Older adults with HIV/AIDS in rural China. Open AIDS J. (2013) 7:51–7. doi: 10.2174/1874613601307010051 24454590 PMC3893720

