# Supplementary material for: HIV testing history and positivity among repeat testers and first-time testers in older adults: a descriptive study from Hejiang county, Southwest China, 2018–2025
Source: Front Public Health. 2026 Jun 19;14:1836119. doi: 10.3389/fpubh.2026.1836119 (PMC13328018; doi:10.3389/fpubh.2026.1836119)
Supplement: Supplementary file 1 [file Table_1.docx]

Supplementary Material

# Supplementary Tables

**Supplementary Table S1. Missing data patterns and comparison of complete and incomplete cases in the baseline negative cohort (n = 214,541)**

| **Variable** | **Missing / Abnormal,**  **n (%)** | **Complete cases**  **n (%)** | **Incomplete cases**  **n (%)** | **P-value** |
| --- | --- | --- | --- | --- |
| Gender | 8 (<0.10) | – | – | – |
| Education | 13,966 (6.51) | – | – | – |
| Marital status | 8,461 (3.94) | – | – | – |
| Age group | 22 (0.01) | – | – | – |
| **Baseline characteristics** |  |  |  |  |
| Male, n (%) | – | 90,426 (45.93) | 8,727 (49.42) | <0.001 ^a^ |
| Total, n (%) ^*^ | – | 196,883 (100.00) | 17,658 (100.00) | - |

^a^ Chi-square test comparing complete vs. incomplete cases for male proportion.

^*^**Individuals may have multiple missing items; total excluded = 17,658.**

**Supplementary Table S2. Missingness in education and marital status by age group in the baseline negative cohort**

| **Age group** | **Total**  **N** | **Education**  **missing**  **n (%)** | **Marital status missing**  **n (%)** |
| --- | --- | --- | --- |
| 50–59 | 65,576 | 6,448 (9.83) | 3,994 (6.09) |
| 60–69 | 60,072 | 4,266 (7.10) | 2,483 (4.13) |
| 70–79 | 64,929 | 2,362 (3.64) | 1,404 (2.16) |
| ≥80 | 23,942 | 888 (3.71) | 578 (2.41) |
| Missing^*^ | 22 | 2 (9.1) | 2 (9.1) |

**^*^Age group missing refers to individuals with no recorded age; they were excluded from all analyses.**

**Supplementary Table S3. Demographic characteristics of the HIV-negative baseline repeat tester cohort, 2018-2024 (N=214,541)**

| **Characteristic** | **N (%)** |
| --- | --- |
| **Gender** |  |
| Male | 99,153 (46.22) |
| Female | 115,380 (53.78) |
| **Age group** |  |
| 50-59 | 65,576 (30.57) |
| 60-69 | 60,072 (28.00) |
| 70-79 | 64,929 (30.27) |
| ≥80 | 23,942 (11.16) |
| **Marital Status** |  |
| Unmarried | 7,013 (3.27) |
| Married | 168,810 (78.68) |
| Widowed | 22,229 (10.36) |
| Divorced | 3,106 (1.45) |
| Unknown ^*^ | 4,921 (2.29) |
| Missing ^*^ | 8,461 (3.94) |
| **Education Level** |  |
| Illiterate or semi-illiterate | 64,656 (30.14) |
| Primary school | 84,755 (39.51) |
| Junior high school | 44,667 (20.82) |
| Senior high school or above | 6,498 (3.03) |
| Missing ^*^ | 13,966 (6.51) |
| **Total** | 214,541 (100.00) |

**^*^** “Unknown” indicates a recorded marital status of “Unknown” (code 90); “Missing” indicates no recorded marital status. Education level was missing for a small proportion of individuals, as shown in the table.
